# Survival of children with trisomy 18 associated with the presence of congenital heart disease and intervention in the Republic of Korea

**DOI:** 10.1186/s12887-023-04056-4

**Published:** 2023-05-20

**Authors:** In Gyu Song, Seung Han Shin, Yoon-Min Cho, Youna Lim

**Affiliations:** 1 Department of Pediatrics, Severance Children’s Hospital, Yonsei University College of Medicine, Seoul, South Korea; 2 Department of Pediatrics, Seoul National University Children’s Hospital, Seoul National University College of Medicine, Seoul, South Korea; 3 Health Insurance Research Institute, National Health Insurance Service, Wonju, South Korea; 4 Graduate School of Public Health, Seoul National University, Seoul, South Korea

**Keywords:** Trisomy 18 syndrome, Survival, Heart defects, congenital, Republic of Korea

## Abstract

**Background:**

Trisomy 18 syndrome (T18) is the second most common autosomal trisomy and has a high risk of fetal loss and stillbirth. Aggressive surgical treatments for the respiratory, cardiac, or digestive systems of patients with T18 were previously futile, while the results of recent studies are controversial. Over the past decade, there have been approximately 300,000 to 400,000 births annually in the Republic of Korea; however, there have been no nationwide studies on T18. This nationwide retrospective cohort study aimed to determine the prevalence of T18 in Korea and its prognosis according to the presence of congenital heart disease and relevant interventions.

**Methods:**

This study utilized NHIS-registered data between 2008 and 2017. A child was defined as having T18 if the ICD-10 revision code Q91.0–3 was reported. Subgroup analysis was performed for children with congenital heart diseases, and survival rates were compared based on the history of cardiac surgical or catheter interventions. The primary outcomes in this study were the survival rate during the first hospitalization period and the 1-year survival rate.

**Results:**

Of the children born between 2008 and 2017, 193 were diagnosed with T18. Of these, 86 died, with a median survival of 127 days. The 1-year survival rate for children with T18 was 63.2%. The survival rate in the first admission of children with T18 who did and did not have congenital heart disease was 58.3% and 94.1%, respectively. Children with heart disease who underwent surgical or catheter intervention had a longer survival time than those who did not.

**Conclusions:**

We suggest these data could be used in ante- and postnatal counseling. Ethical concerns about the prolonged survival of children with T18 remain; however, the potential benefits of interventions for congenital heart disease in this population need further study.

**Supplementary Information:**

The online version contains supplementary material available at 10.1186/s12887-023-04056-4.

## Background

Trisomy 18 syndrome (T18) is the second most common autosomal trisomy and has a high risk of fetal loss and stillbirth. The prevalence in live births is estimated as 1/6,000–1/8,000. However, the actual prevalence may be higher (1/2,500–1/2,600) because of the high risk of fetal death and pregnancy termination after prenatal diagnosis [1]. This syndrome is closely associated with preterm delivery, congenital malformations, feeding and breathing difficulties, and high neonatal mortality rates (40–60%) [2, 3]. Recent two population-based studies from the United States (US) have reported the median survival of T18 as 7–8 days, with a 1-year survival rate of 3.0–13.4% [4, 5]. Therefore, aggressive respiratory, cardiac, and digestive system surgical interventions have been regarded as futile by clinicians and parents of children with T18 until the early 2000s.

In contrast, some reports have indicated that the survival rate of children with T18 may increase while receiving active treatment. Data of vital statistics from Japan demonstrated that more surgical interventions provided during 1995–2016 increased the median survival time of children with T18 from 28 to 54 days [3]. A multicentre registry study in the US reported that patients who received cardiac intervention and were discharged from the hospital alive had a median survival rate of 16.2 years [6]. However, decisions for active treatment of T18 are challenging for families and healthcare professionals, as complex ethical issues often emerge during treatment because of their poor prognosis and the need for constant medical support throughout their lives [7].

In 2019, the Constitutional Court in the Republic of Korea (Korea) determined the anti-abortion law unconstitutional officially. Until then, abortion due to fetal disease was illegal [8]. The government started to amend the law, and medical professionals were asked to suggest the criteria for abortion. As the law is revised, the frequency of antenatal consultation for fetal diseases may increase. Therefore, establishing reliable clinical prognoses for this life-limiting condition is required to provide information during antenatal and postnatal consultation. In the last decade, there have been 300,000–400,000 annual births in Korea; however, there have been no nationwide studies on the epidemiology and outcomes of T18. Therefore, this nationwide retrospective cohort study aimed to determine the prevalence of T18 in Korea and its outcomes using the National Health Insurance System (NHIS) database.

## Methods

### Data source

NHIS data containing demographics, healthcare utilization, and diagnoses based on the International Classification of Diseases, 10th revision (ICD-10), were used in this study. Several studies utilizing the Korean NHIS cohorts have been conducted, and more recently, studies on the long-term outcomes of neonates using the NHIS database have been published [9–12]. This study was designed and performed according to the principles of the Declaration of Helsinki. The Seoul National University Hospital institutional review board exempted this study from deliberation (E-2207-050-1339) due to its retrospective nature, as it utilized anonymized NHIS data. Permission to access all the data of people born between 2008 and 2017 was provided by the NHIS review committee.

### Study population and variables

A child was defined as having T18 if the ICD-10 codes (Q91.0, Q91.1, Q91.2, and Q91.3) were reported during an inpatient admission or visit to outpatient clinics. Follow-up observations of these infants were conducted until December 31, 2018. The type of insurance (national health insurance or medical aid) and the NHIS premium depending on income levels were employed as proxies for measures of income level. The lowest income category was designated as those receiving medical aid. In addition, the NHIS group was split into four groups: category 1, < 25% premium; category 2, 25–50% premium; category 3, 50–75% premium; and category 4, > 25% premium. We reviewed and ranked all ICD-10 codes associated with congenital malformation, deformation, and chromosomal abnormalities (Q codes). A child was defined as having a history of congenital heart disease (CHD) if diagnosed with Q20–26. Heart anomalies that usually regress spontaneously, such as atrial septal defect (ASD) (Q21.1) and patent ductus arteriosus (PDA) (Q25.0), were excluded from CHD codes. Among CHDs, the annual number of children who underwent surgical or catheter intervention (intervention) for CHDs was counted using health insurance claim codes (eTable 1 in Supplement 1). Additionally, the number of children with T18 who had tracheotomy or gastrostomy was counted. The mortality rate was analyzed in two categories. We examined mortality during the first hospitalization period and before 1 year after birth.

### Statistical analysis

The study employed Pearson’s chi-square analysis to compare the mortality rates between groups according to the history of cardiac intervention. Kaplan-Meier survival analysis and log-rank tests were utilized to describe long-term survival and compare groups, respectively. In this study, we used a two-sided test with a significance level of p < 0.05 to determine statistical significance. All analyses were performed using SAS (version 9.4; SAS Institute Inc., Cary, NC, USA).

## Results

**Fig. 1:**
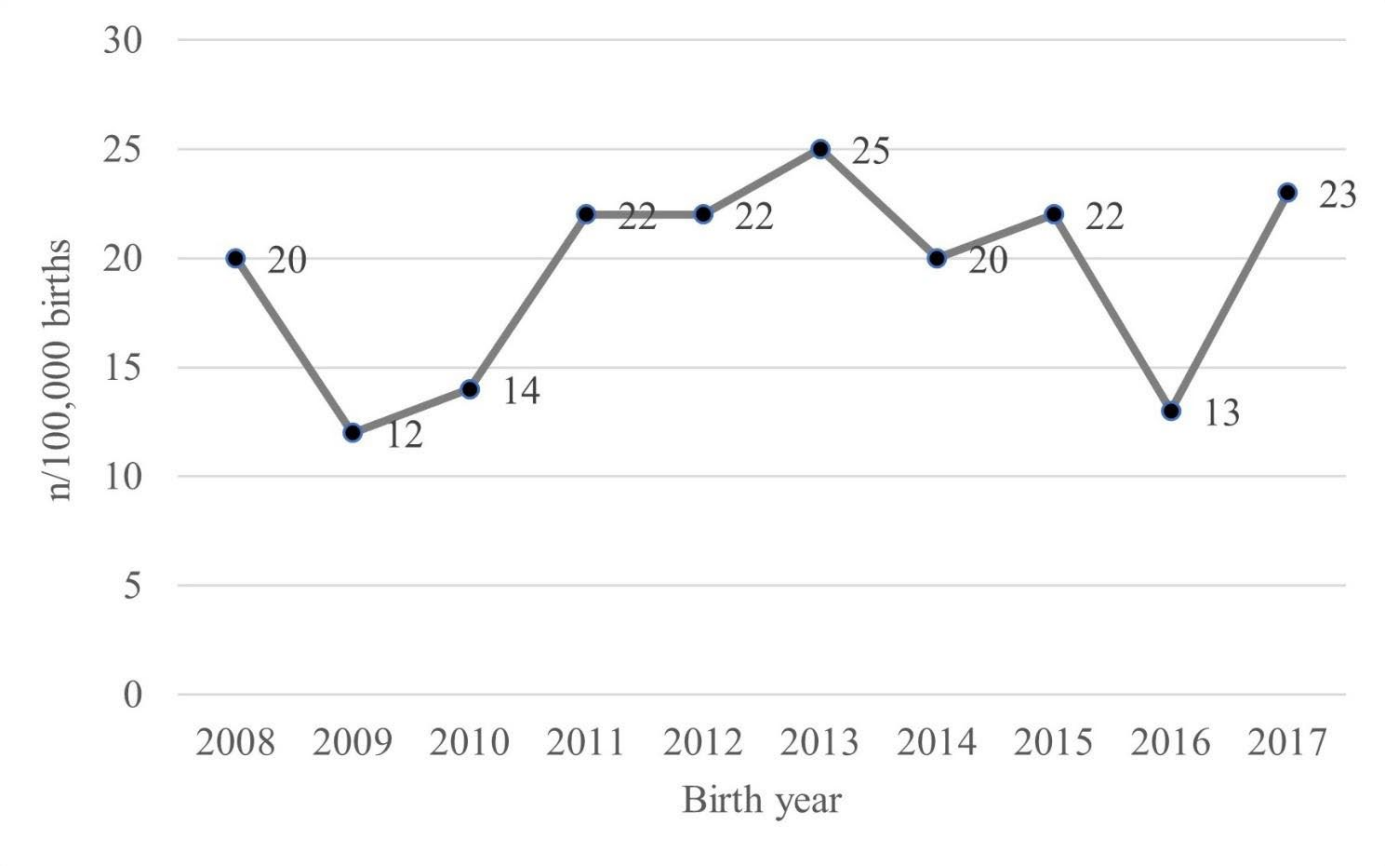
Annual number of birth of children with trisomy 18 per 100,000 birth in the Republic of Korea

**Fig. 2:**
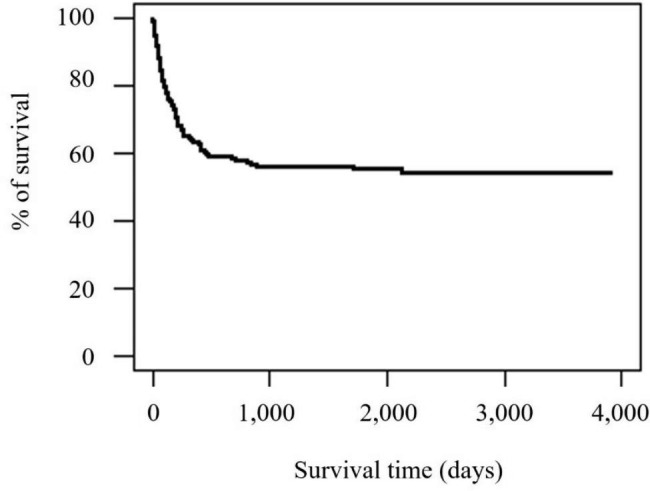
Survival curve for children with trisomy 18 in the Republic of Korea

The NHIS database included 193 children diagnosed with T18 who were born between 2008 and 2017. Annually, 12–25 children are diagnosed with T18 (2.7–6.4 per 100,000 births) (Fig. 1). Of these, 86 children died, with a median survival of 127 days. There was no discernible trend in survival rate during the study period. Among these mortality cases, 48 (24.9%) and 71 (36.8%) children died during the first admission and their first year of life, respectively (Tables 1 and 2; Fig. 2). Tracheotomy and gastrostomy were performed in 14 children at a median of 129 and 175 days after birth, respectively (Table 1).

**Table 1:** Demographics of children with trisomy 18 in the Republic of Korea (n, %)

Total number		193 (100)
Birth year	2008	20
	2009	12
	2010	14
	2011	22
	2012	22
	2013	25
	2014	20)
	2015	22
	2016	13
	2017	23
Sex	Female	120 (62.2)
Income	Medical aid + 1st quartile	29 (15.0)
	2nd quartile	44 (22.8)
	3rd quartile	75 (38.9)
	4th quartile	45 (23.3)
Surgery	Heart surgery	51 (26.4)
(Median age at surgery (1st and 3rd quartile))	Age at surgery	38 (6, 70) days
	Tracheotomy	14 (7.3)
	Age at surgery	129 (93, 419) days
	Gastrostomy	14 (7.3)
	Age at surgery	175 (0, 380) days
Death^a^	During 1st admission	48 (24.9)
	Age at death	77 (31.5, 166.5) days
	Within 1st year of life	71 (36.8)
	Age at death	95 (50, 195) days

a. Number of deaths up to the observation period at the end of 2018 and the median age at death (1st and 3rd quartile)

**Table 2:** Annual incidence of trisomy 18 and its health outcomes in Korea (n, %)

	2008	2009	2010	2011	2012	2013	2014	2015	2016	2017	Total
Trisomy 18	20	12	14	22	22	25	20	22	13	23	193
Congenital heart disease	13 (65.0)	5 (41.7)	9 (64.3)	13 (59.1)	9 (40.9)	13 (52.0)	12 (60.0)	13 (59.1)	7 (53.8)	14 (60.9)	108 (56.0)
Cardiac intervention among CHD ^a^	1 (7.7)	0 (0)	7 (77.8)	9 (69.2)	4 (44.4)	7 (53.8)	6 (50.0)	3 (23.1)	3 (42.9)	7 (50.0)	47 (43.5)
Tracheotomy	0 (0)	1 (8.3)	1 (7.1)	2 (9.1)	1 (4.6)	1 (4.0)	3 (15.0)	1 (4.6)	0 (0)	4 (17.4)	14 (7.3)
Death^b^	10 (50.0)	5 (41.7)	8 (57.1)	7 (31.8)	6 (27.3)	11 (44.0)	12 (60.0)	11 (50.0)	7 (53.8)	9 (39.1)	86 (44.5)

a. Congenital heart disease

b. Number of deaths up to the observation period at the end of 2018

The most common congenital disease codes that children with T18 had were ‘congenital malformations of cardiac septa (Q21)’, which includes ASD and ventricular septal defect; ‘congenital malformations of great arteries (Q25)’, which subsume PDA; ‘congenital deformities of feet (Q66)’; and ‘other congenital malformations of the brain (Q04)’ (eTable 2 in Supplement 1). Except for PDA and ASD, the ventricular septal defect was the most common CHD, followed by coarctation of the aorta and double outflow of the right ventricle (eTable 3 in Supplement 1). Consequently, 108 (56.0%) children with T18 were diagnosed with CHDs, and 47 (43.5%) received cardiac intervention. In 2008 and 2009, few children with T18 underwent heart intervention; however, 23.1–77.8% of children with T18 and CHD underwent intervention from 2010. Only one or two infants underwent a tracheotomy yearly; however, approximately more infants underwent surgery in 2014 and 2017 (3 and 4, respectively).

**Fig. 3:**
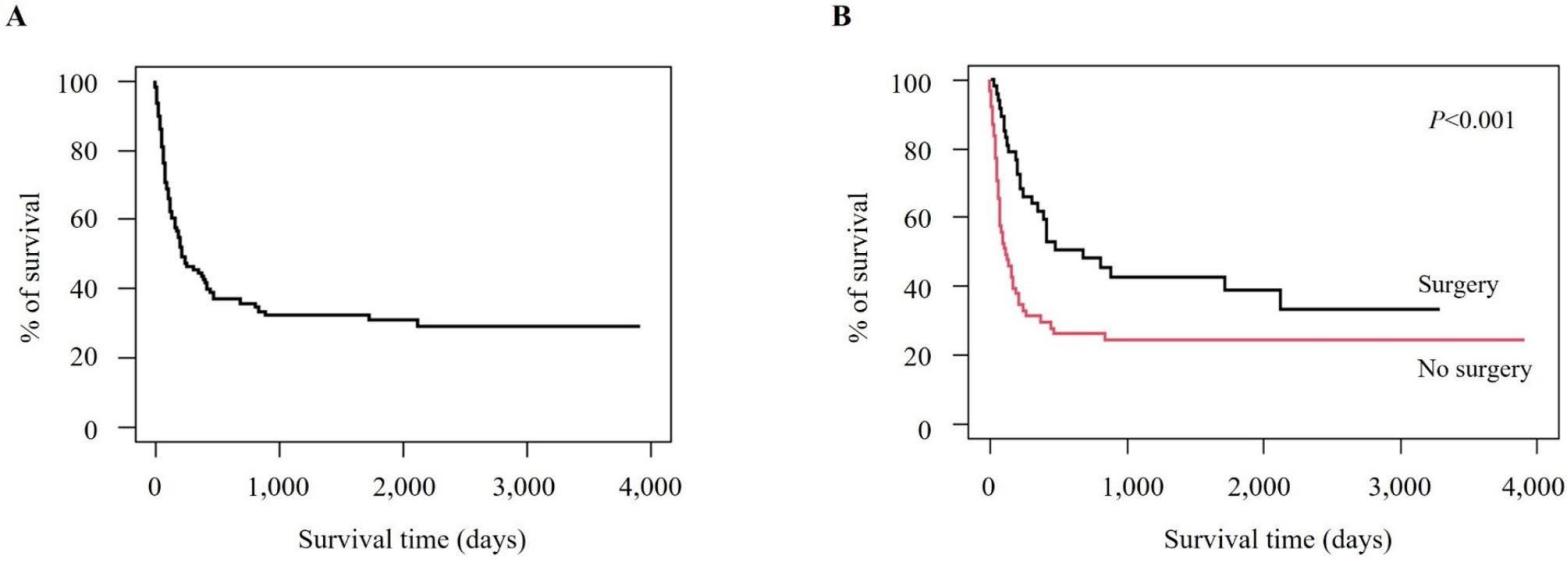
Survival curve for children with trisomy 18 and congenital heart disease in the Republic of Korea. (**A**) Survival curve for children with trisomy 18 and congenital heart disease. (**B**) Survival curve and log-rank test for children with trisomy 18 and congenital heart disease based on a history of heart surgery

Notably, 94.1% of children without CHD survived until discharge from the first hospitalization. Among patients with heart disease, the survival rate during the first admission for children who underwent intervention was higher than for those who did not (70.2% vs. 49.2%, *P* = 0.028) (Table 3), with significantly longer survival days (686 days vs. 123 days, *P* < 0.001) (Fig. 3).

**Table 3:** The survival rate of trisomy 18 in the Republic of Korea

The survival rate of trisomy 18 during the first admission							
Congenital Heart Disease				Cardiac intervention			
	Yes	No	*P*-value		Yes	No	*P*-value
Survive	63 (58.3)	80 (94.1)	< 0.001	Survive	33 (70.2)	30 (49.2)	0.028
Death	45 (41.7)	5 (5.9)		Death	14 (29.8)	31 (50.8)	
Total	108	85		Total	47	61	

## Discussion

This nationwide population-based study was the largest on intervention and outcomes of children with T18 in Korea. The median survival time of mortality cases was 127 days and the 1-year survival rate was 63.2%, with no evident change in the survival rate for 10 years. Among 108 children with CHD, 47 (43.5%) underwent cardiac intervention, and their survival days were longer than those who did not undergo the intervention.

In this study, 193 children were diagnosed with T18 over 10 years (0.4 per 10,000 births), a lower number than in previous studies conducted in other countries. Approximately 1.5 per 10,000 live births in the US were affected by T18, and multinational research reported that the mean prevalence of T18 among live births was 1.07 (95% CI 0.77, 1.38) per 10,000 births [2, 6]. The low number of births with T18 may be attributed to the relatively higher rate of induced abortion in Korea [13, 14].

The median survival time (25–75 percentiles) of mortality cases was 127 (57, 262) days in our study, which was longer than those in previous studies with similar study periods. A recent study from Japan has reported that the median survival time of children with T18 who were born between 2006 and 2016 was 54 (1–196) days [3]. In another study that analyzed data from children with T18 who were born in the greater Cincinnati area between 2012 and 2018, the median length of survival was 29 days [15]. This phenomenon could be partly explained by more active treatment in Korea. Compared to a population-based study in the United States, the rates at which children with T18 received cardiac intervention and tracheotomy in this study were higher. In the United States study, 7.1% underwent cardiac surgery, and 4.5% underwent tracheostomy [16], whereas, in this study, it was 24.3% (n = 47) and 7.3%, respectively. In the United States, the average age for cardiac procedure and tracheotomy was 0.7 and 1.9 years, respectively. However, in this study, the median ages for these surgeries were 38 days and 129 days after birth, respectively [16]. The direct comparison between the two studies might be inappropriate as the measured values (average and median) were different, and there was a lack of data on the preoperative status of the patients in both studies. However, it was observed that procedures in Korea were relatively performed in the early life stages. Two single referral center studies conducted in Korea reported an increasing proportion of children with T18 receiving active treatment after birth [17, 18]. In one study, positive pressure ventilation and intubation were performed in 79.2% and 54.2% of children with T18, respectively [17]. Furthermore, another study reported that 77.2% were intubated, higher than those in previous studies [18]. Several studies, including the aforementioned Korean single-center studies, have discovered that active intervention in children with T18 increased survival [17–21]. Parents who decide to give birth despite the high rate of abortion in Korea may prefer active interventions for their affected infant. Additionally, as pediatric palliative care was unavailable in Korea until 2017 and advance care planning was not conducted actively, intensive treatment may have been conducted according to the doctors’ intentions [22, 23].

Our study included 108 children with T18 and CHD. In previous studies, there has been debate about the association between CHD and the survival of T18 [24]. However, congenital heart disease is generally known to be a major cause of mortality in T18 [25]. In this study, we observed T18 with ventricular septal defect, coarctation of the aorta, atrioventricular septal defect, and a few cases with complex heart disease (data not shown). Thease heart diseases can be a contributing factor to mortality and the risk may increase if left untreated. Among the patients with T18 and congenital heart diseases, 43.5% underwent cardiac intervention, and the survival rate was higher in this group. This finding still needs cautious interpretation because parents of children with T18 and severe heart disease may have denied receiving active treatment and only desired to provide comfort care for their infants. In a single-center study of prenatal counselling and parental decision-making after a fetal diagnosis of trisomy 13 or 18 from the US, more than half of the families decided to terminate the pregnancy. In contrast, only 2% of the family desired to provide active treatment [26]. Nevertheless, the result of this study was concordant with recent studies reporting that cardiac intervention prolonged survival in children with T18 [6, 27, 28]. When intervention is anticipated to improve patient outcomes, those interventions should be one of the options during the discussion for the patient’s best interest.

Severe developmental delay, long-term dependence on medical equipment, and survival have been considered in deciding on a treatment policy for T18 patients. However, several studies on the quality of life of children with T18 have further indicated that considering the family’s perspective and knowledge of natural history when formulating policies for T18 treatment is crucial. Parents of patients with T18 or trisomy 13 syndrome patients reported that their child was happy and enriched their family despite their children’s severe conditions [29]. In another study in Japan, parents of patients with T18 reported that their children interacted with their parents and siblings throughout their lives, resulting in quality family time, and that the parents were positive about raising their kids [30]. Moreover, parents of patients with T18, who underwent cardiac surgery, rated their child’s quality of life as ‘high.’ Qualitative analysis revealed a profound understanding of the child’s relationality and valued life meaning [31]. Qualitative studies on the quality of life of T18 patients and their families in Korea are required.

This study had some limitations. First, in this study, the observation period varied from 1 year to 11 years due to the children’s different birth years, and ended in December 2018. Although we attempted to mitigate this issue by using a survival curve, there were still limitations to this approach. We acknowledge the limitations of our analysis due to the absence of information on the number of patients at risk at each time point and censoring marks. We had access to this information at the time of analysis, but unfortunately overlooked it and are now unable to retrieve the data. Thus, our presented survival curve may not provide a comprehensive view of the survival outcomes, and readers should interpret our results with caution. Second, we did not have patient information on T18 mosaicism. Patients with T18 mosaicism revealed an extremely variable phenotype; they occupy a small portion of the disease group and may not affect the study’s result [1]. Third, as detailed medical history could not be obtained from national health insurance data, the severity of the disease could not be considered when cardiac interventions were determined. Owing to the nature of the study, there was no data on the decisions made by the clinicians and parents regarding active treatment or comfort care of the patient. These limitations can be overcome by conducting a cohort study and establishing a registry in the future. This is the first study identifying the nationwide prevalence of T18 in Korea and its prognosis. It appears improbable that many children with T18 were omitted from the analysis because approximately 99% of births in Korea occur in hospitals, and physicians have to register ICD-10 codes for reimbursement of medical costs [32]. In addition, the number of children with T18 who died and were identified in this study between 2010 and 2015 (55 children) represented 85.9% of the national death statistics data (64 children), and it was 97.6% between 2012 and 2015 [33].

## Conclusion

In this study, fewer children with T18 were reported in the NHIS database in Korea than in other countries; however, many of them received active treatments and had longer survival rates. Recently, advance care planning has been implemented, and the laws regarding abortion have been amended in Korea. Due to these social and legal changes, more ante- and postnatal consultation on T18 treatment are expected, and patient-centered care will be more warranted. Therefore, we expect these data to help determine the disease prognosis and find the best interests for children with T18.

## Electronic supplementary material

Below is the link to the electronic supplementary material.

Supplementary Material 1

## Data Availability

The data that support the findings of this study are available from NHIS but restrictions apply to the availability of these data, which were used under license for the current study, and so are not publicly available. Data are however available from the authors upon reasonable request and with permission of NHIS.

## List of Abbreviations

ASD: Atrial septal defect
CHD: Congenital heart disease
NHIS: National Health Insurance System
PDA: Patent ductus arteriosus
T18: Trisomy 18 syndrome
