# Supplementary material for: Survival of children with trisomy 18 associated with the presence of congenital heart disease and intervention in the Republic of Korea
Source: BMC Pediatr. 2023 May 20;23:252. doi: 10.1186/s12887-023-04056-4 (PMC10199514; doi:10.1186/s12887-023-04056-4)
Supplement: Supplementary file 1 — Supplementary Material 1 [file 12887_2023_4056_MOESM1_ESM.docx]

| eTable 1. Health insurance claim codes for counting the annual number of children who underwent surgical or catheter intervention for congenital heart diseases. | |
| --- | --- |
| **Codes** | **Surgical or catheter intervention** |
| O1671 | Closure of Patent Ductus Arteriosus - Ligation |
| O1672 | Closure of Patent Ductus Arteriosus - Division |
| O1680 | Correction of Coarctation of Aorta |
| O1701 | Shunt Procedure |
| O1703 | Pulmonary Artery Banding |
| O1710 | Operation of Atrial Septal Defect |
| O1711 | Operation of Atrial Septal Defect - Minimally Invasive Surgery |
| O1721 | Operation of Ventricular Septal Defect - Congenital |
| O1723 | Operation of Ventricular Septal Defect - Congenital (Minimally Invasive Surgery) |
| O1730 | Open Mitral Commissurotomy |
| O1740 | Operation of Aortic Stenosis |
| O1750 | Operation of Pulmonary Stenosis |
| O1760 | Operation of Tricuspid Stenosis |
| O1770 | Operation of Atrial Septal Defect And Pulmonary Valvular Stenosis |
| O1800 | Total Correction of Tetralogy of Fallot |
| O1791 | Valve Replacement - Tricuspid Valve |
| O1792 | Valve Replacement - Mitral Valve |
| O1793 | Valve Replacement - Aortic Valve |
| O1794 | Reoperation of Valvuloplasty - Tricuspid Valve |
| O1795 | Reoperation of Valvuloplasty - Mitral Valve |
| O1796 | Reoperation of Valvuloplasty - Aortic Valve |
| O1797 | Valve Replacement - Pulmonary Valve |
| O1798 | Reoperation of Valvuloplasty - Pulmonary Valve |
| O1810 | Operation of Ventricular Septal Defect And Pulmonary Valvular Stenosis |
| O1821 | Repair of Endocardial Cushion Defect - Partial |
| O1822 | Repair of Endocardial Cushion Defect - Complete |
| O1825 | Left Ventricular Outflow Track Augmentation |
| O1826 | Right Ventricular Outflow Track Reconstruction |
| O1840 | Repair of Ruptured Aneurysm of Sinus Valsalva |
| O1850 | Repair of Complicated Congenital Heart Diseases |
| O1861 | Left And Right Pulmonary Artery Reconstruction |
| O1873 | Functional Correction of Single Ventricle - Glenn Operation |
| O1874 | Functional Correction of Single Ventricle - Fontan Operation |
| O1875 | Rastelli's Operation |
| O1878 | Repair of Total Anomalous Pulmonary Venous Return |
| O1879 | Repair of Transposition of Great Arteries |
| O1981 | Resection of Atrial Myxoma |
| O1982 | Resection of Cardiac Tumor - Others |
| OZ751 | Percutaneous Closure of Interatrial Septal Defect |
| M6510 | Percutaneous Closure of Patent Ductus Arteriosus |
| M6521 | Percutaneous Atrial Septostomy - Balloon |
| M6522 | Percutaneous Atrial Septostomy - Blade |
| M6531 | Percutaneous Valvuloplasty - Mitral Valve |
| M6532 | Percutaneous Valvuloplasty - Aortic Valve |
| M6533 | Percutaneous Valvuloplasty - Pulmonic Valve |
| M6595 | Percutaneous Transluminal Angioplasty - Aortic |
| M6596 | Percutaneous Transluminal Angioplasty - Pulmonary |
| M6603 | Percutaneous Intravascular Installation of Metallic Stent - Aortic |
| M6604 | Percutaneous Intravascular Installation of Metallic Stent - Pulmonary |
| M6611 | Percutaneous Intravascular Installation of Stent Graft - Aortic |

| eTable 2. Most common congenital diseases in trisomy 18 | | |
| --- | --- | --- |
| ICD-10^a^ | Disease category | n (%) |
| Q21 | Congenital malformations of cardiac septa | 123 (63.7) |
| Q25 | Congenital malformations of great arteries | 98 (50.8) |
| Q66 | Congenital deformities of feet | 22 (11.4) |
| Q04 | Other congenital malformations of the brain | 21 (10.9) |
| Q24 | Other congenital malformations of the heart | 18 (9.3) |
| Q74 | Other congenital malformations of limb(s) | 17 (8.8) |
| Q03 | Congenital hydrocephalus | 15 (7.8) |
| Q79 | Congenital malformations of the musculoskeletal system | 15 (7.8) |
| Q63 | Other congenital malformations of the kidney | 14 (7.3) |
| Q10 | Congenital malformations of the eyelid, lacrimal apparatus, and orbit | 13 (6.7) |
| Q53 | Undescended testicle | 13 (6.7) |
| Q62 | Congenital obstructive defects of the renal pelvis and congenital malformations of the ureter | 12 (6.2) |
| Q89 | Other congenital malformations | 12 (6.2) |
| Q20 | Congenital malformations of cardiac chambers and connections | 11 (5.7) |
| Q31 | Congenital malformations of the larynx | 11 (5.7) |
| Q95 | Balanced rearrangements and structural markers | 11 (5.7) |
| Q35 | Cleft palate | 10 (5.2) |
| Q42 | Congenital absence, atresia, and stenosis of the large intestine | 10 (5.2) |
| Q75 | Other congenital malformations of skull and face bones | 10 (5.2) |

^a^International Classification of Diseases, 10th revision

| eTable 3. Ten most common congenital heart diseases in trisomy 18 | | |
| --- | --- | --- |
| ICD-10^a^ | Disease | n (%) |
| Q250 | Patent ductus arteriosus | 93 (48.2) |
| Q210 | Ventricular septal defect | 89 (46.1) |
| Q211 | Atrial septal defect | 82 (42.5) |
| Q251 | Coarctation of aorta | 14 (7.3) |
| Q249 | Unspecified malformation | 12 (6.2) |
| Q201 | Double outlet right ventricle | 10 (5.2) |
| Q256 | Pulmonary artery stenosis | 7 (3.6) |
| Q212 | Atrioventricular septal defect | 6 (3.1) |
| Q231 | Aortic valve insufficiency | 4 (2.1) |
| Q240 | Dextrocardia | 1. (2.1) |

^a^International Classification of Diseases, tenth revision
